# Evolutionary game analysis of data sharing among large and medium-sized enterprises in the perspective of platform empowerment

**DOI:** 10.1038/s41598-024-62156-2

**Published:** 2024-05-20

**Authors:** Dan Li, Xudong Mei

**Affiliations:** School of Business Administration, Liaoning Technical University, Xingcheng, 125100 China

**Keywords:** Digital platform, Enterprises, Data sharing, Digital economy, Evolutionary game, Mathematics and computing, Applied mathematics, Computational science, Information technology

## Abstract

With the swift advancement of the global digital economy, data has emerged as a critical component in fostering the integration of large enterprises with small and medium-sized enterprises (SMEs). Nevertheless, due to disparities in resources and capabilities between these entities, there is a deficiency in the willingness to share data, hindering the full actualization of data’s potential value. Hence, it is imperative to facilitate a novel cooperative development paradigm wherein platforms enable data sharing among large enterprises and SMEs. In this paper, we construct a tripartite evolutionary game model encompassing large enterprises, SMEs, and digital platforms, examine the evolutionary stable strategies adopted by these entities in the data sharing, and use numerical simulation to explore the system’s evolutionary stability under various influencing factors. Contrasting with prior research, this study considers the heterogeneity of enterprise scale and delves into the data sharing dynamics between large enterprises and SMEs. Simultaneously, it positions the digital platform as a player in the game, examining its impact on data sharing among the enterprises. Findings indicate that: (1) SMEs exhibit greater eagerness for data sharing compared to large enterprises, which display a U-shaped influence during the process; (2) Digital platforms are particularly sensitive to costs, with the platform’s initiative and the service quality will affect enterprises strategic choices; (3) Government subsidies positively encourage tripartite cooperation, and robust data security governance framework is crucial for enterprises. Finally, based on the results of the study and combining with the current situation of digital economy development, it puts forward the suggestions for promoting platforms to empower large enterprises and SMEs to realize data sharing and the prospects for future research.

## Introduction

In the context of the complex and changing world environment, the global industrial chain supply chain is facing the challenge of insecurity and instability, and the coordination of upstream and downstream support of the industrial chain in various countries has become more difficult. As the main body of the industrial and supply chain, large enterprises and small and medium-sized enterprises (SMEs) play different roles and work together for the stability and development of the industrial and supply chain. Therefore, the synergistic development of large enterprises and SMEs is crucial for enhancing the overall security and adaptability of national industrial and supply chains^1,2^. However, the synergistic development of SMEs and large enterprises has not received adequate attention. Both types of enterprises often lack the motivation for cooperative growth and are bereft of efficient mechanisms to foster effective partnerships. In reality, While SMEs are the foundation of the global economy, constituting 90% of all businesses and contributing nearly 70% of global jobs and GDP^3^, disparities in resources and capabilities between them and large enterprises significantly hinder their synergistic development. Internally, it is difficult for SMEs to establish links with large enterprises to obtain resources due to their lack of innovation capital and talents, as well as their weak knowledge and technology base. Although large enterprises have stronger overall strength and objective conditions to assist SMEs in overcoming these deficiencies, concerns over the spillover of their “private property” such as internal core knowledge and technology deter them from offering substantial support. Furthermore, external factors such as market competition, imperfect government policies, and uncertainty risks impede the cooperative progress between large enterprises and SMEs. Traditional cooperative development models and tools are difficult to meet the demand for efficient cooperation among enterprises in the digital era, resulting in the sluggish cooperative development. Therefore, it is of great theoretical and practical significance to investigate how to promote the cooperative development of large enterprises and SMEs in the digital context.

With the progression of the digital revolution, the digital economy has become the “new engine” of economic growth across various countries. The World Bank estimates that the digital economy contributes to more than 15% of GDP, and in the past decade it has been growing at two and a half times faster than physical world GDP^4^. The rapid development of digital economy not only creates new tools (such as digital platform) for the coordinated development of large enterprises and SMEs, but also creates a new model (platform ecological model) for their coordinated development. However, realizing this necessitates leveraging data’s driving role. As a new production factor, data itself can’t generate value, and its value realization significantly differs from traditional resources. Only by collecting, sorting, analyzing and verifying scientifically can we get correct laws and valuable information from data. In this process, it is necessary to dismantle data fragmentation to facilitate comprehensive sharing among different entities, thereby maximizing the value of data through extensive cross-comparison^5^. Many governments have prioritized data sharing as an important development strategy. For example, the European Commission’s *Study on Data Sharing between Companies in Europe* identifies data sharing among enterprises as a key aspect for advancing Europe’s digital economy. Concurrently, some industry giants, such as Airbus and Walmart, have built their proprietary data sharing platforms, which, by sharing data with SMEs along the industrial and supply chains, collectively enhance production efficiency, product quality, service levels, and the stability of these chains. As an important data infrastructure, platforms have strong resource connection ability and data processing ability^6^. By offering digital services, they can horizontally link large enterprises and SMEs and vertically integrate various departments, providing flexible digital capabilities while improving data sharing efficiency and quality among enterprises, fostering cooperation, reducing information asymmetry^7^, and improves economic benefits^8^. Therefore, studying how to play the role of platform empowerment and promote data sharing among large enterprises and SMEs is not only conducive to their coordinated development and the enhancement of the stability of the industrial and supply chain, but also for releasing the value of data and creating greater benefits for enterprises.

Scholars have extensively explored data sharing among enterprises, primarily focusing on three dimensions: sharing value, influencing factors, and realization methods along with incentive mechanisms. (1) Sharing value. Wang et al. (2022), Aben et al. (2021) and Chen et al. (2021) all found in their studies that data and information sharing among enterprises can reduce information asymmetry and supply chain costs^9–11^. Han et al.^12^ constructed an econometric model using Chinese manufacturing firms’ data and demonstrated that data sharing has a significant positive impact on firm productivity. Wang et al.^13^ pointed out that data sharing increase the probability and scale of innovation investment of enterprises. Franco et al.^14^ found that information sharing is conducive to enhancing the cooperative ability between enterprises, expanding the scale effect, and subsequently improving the Pareto efficiency through a field survey of enterprises. There is a consensus among scholars on the importance of data sharing among enterprises. (2) Influencing factors. Du et al.^15^ pointed out that enterprise relationship have a significant effect on the willingness of inter-firm data sharing. Quach et al.^16^ proved the impact of data risk on enterprise data sharing. Feng et al.^17^ from the perspective of government behavior, found that government incentive policies can influence information sharing among platform enterprises. Guo et al.^18^ argued that data sharing may diminish enterprises’ competitiveness , leading to a decline in their willingness to share data. (3) Realization modes and incentives. Lee et al.^19^ classified data sharing into three categories: information transmission mode, third-party mode, and information center mode. Zhong et al.^20^ comparatively analyzed differences in information sharing modes between manufacturers with or without platform intervention and disparities in information sharing models between retailers. Guan et al.^21^ proposed a data sharing incentive strategy based on a two-part compensation contract around the supply chain demand uncertainty issues. Lu et al.^22^ compared two incentive strategies—revenue sharing and fixed compensation—and analyzed their impact on provider information sharing through a principal-agent model. Existing researches provide a rich theoretical foundation for enterprise data sharing, but most of them However, most studies generalize both large enterprises and SMEs, overlooking the differences’ impact on data sharing. Therefore, the influencing factors and mechanisms of data sharing between large enterprises and SMEs warrant further exploration.

In the past, enterprises usually use manual entry, script processing, traditional tools to achieve data sharing. However, these methods are inadequate in the digital era, as they fail to meet the needs of modern businesses and present significant drawbacks^23^. Firstly, data sources vary, making it impossible to manage and convert database tables, files, and interfaces uniformly. Secondly, enterprise business systems are independently constructed, leading to the formation of data islands that hinder timely data sharing among enterprises. Thirdly, there is no guarantee mechanism for data security, precluding global monitoring and analysis of data risks. Most scholars believe that information technology (IT) plays a crucial role in addressing these issues. Anandhi^24^ highlighted that IT infrastructure serves as the starting point for information transfer, expanding its scope and enabling timely, efficient information sharing. Yu et al.^25^ argued that deploying advanced information technology in supply chain systems enhances coordination between upstream and downstream operations, reducing transaction costs among node enterprises. Prajogo et al.^26^ noted that network-based information technology facilitates real-time integration and sharing of inventory planning, demand forecasting, and order scheduling information among supply chain enterprises, supporting core companies in balancing supply and demand throughout the supply chain network. It can be seen that IT provides technical support for the improvement of information sharing level. For risks such as leakage and loss in data sharing, the emergence of blockchain technology provides new ideas and solutions for secure data sharing^27^. Yu et al. and Ma et al.^28,29^ both pointed out that blockchain technology ensures data integrity and resistance to tampering when individual or multiple nodes face attacks through distributed storage, thereby reducing the risk of data leakage. In the digital era, digital platforms as fusion of technology, aggregated data, empowering the application of institutional digital services hubs^30^. They are compatible with various data formats and provide data risk monitoring, constituting an integrated, secure, and efficient data sharing infrastructure encompassing data collection, storage, processing, analysis, and application. Digital platforms contribute significantly to solving efficiency, privacy protection, reliability, cost, and other data sharing issues. However, scholars have not yet fully explored the specific mechanism of how digital platforms can be applied in data sharing between large enterprises and SMEs, which still needs to be further researched.

The essence of the data sharing problem lies in the game of data resources between different subjects, based on the comprehensive consideration of cost and benefit, to find the equilibrium solution that each subject can cooperate well. The traditional game method is based on the assumption of rationality, which has certain limitations. The emergence of evolutionary game theory relaxes the assumption that the participating subjects are all limited rational, which can clearly reflect the dynamic process of the continuous adjustment of their strategies over time. This provides a better research paradigm for analyzing the evolutionary law of economic entities^31,32^. Many scholars began to explore the issue of data sharing based on evolutionary game method. Liu et al.^33^ built an evolutionary game model of data sharing between logistics platforms and suppliers, identifying factors such as agency fees that impact cooperation. Wei et al.^34^ built an evolutionary game model of data sharing among enterprises under government supervision, and used numerical simulation analysis to find that government supervision has little influence on enterprise sharing behavior. Li^35^ based on the evolutionary game model, examined the influence of platform information security on information sharing among enterprises. It can be seen that evolutionary game theory has some research in data sharing, which lays a theoretical foundation for this paper, but at present there are almost no scholars to explore how to promote data sharing between large enterprises and SMEs from the perspective of evolutionary game theory.

In summary, enterprise data sharing has become a focal point of both theoretical and practical attention within the context of digitalization. However, current academic research on the factors that influence the willingness of large enterprises and SMEs to share data is insufficient. Furthermore, there is a paucity of discussion on the impact of digital platforms on data sharing between these entities. On the other hand, although some scholars have designed and optimized the data sharing model among enterprises, the analysis of multi-agent game in data sharing is still insufficient. Ignoring the influence of the differences between large enterprises and SMEs on their data sharing willingness. In view of these research gaps, this paper aims to explore the differences in data sharing between large enterprises and SMEs, as well as the role of digital platforms in promoting data sharing among them. And adopt the evolutionary game method to analyze the influencing factors and dynamic evolution path of data sharing among large enterprises and SMEs under the participation of digital platforms, to elucidate the realization mechanism of data sharing among large enterprises and SMEs. It provides theoretical guidance and practical suggestions for establishing efficient data sharing cooperation between large enterprises and SMEs.

Compared with previous literature related to inter-enterprise data sharing, the innovations of this paper are mainly manifested in three aspects: (1) It considers the heterogeneity between large enterprises and SMEs while examining the issue of enterprise data sharing, which breaks through the limitations of previous studies that categorize large enterprises and SMEs as homogeneous enterprises. This expands the research perspective of the enterprise data sharing problem. (2) It investigates the role of digital platforms in data sharing between large enterprises and SMEs, and based on the evolutionary game theory, an evolutionary game model with digital platforms, large enterprises and SMEs as the main body is constructed. This enriches the application of evolutionary game theory within data sharing research. (3) Utilizing numerical simulation, it analyzes the impact and dynamic evolution trajectory of platform service quality, data sharing benefits, costs, subsidies, and risks on the data sharing willingness of large enterprises and SMEs, and further elucidates the factors and mechanisms that influence enterprise data sharing.

This study identifies distinct characteristics in the steady-state data sharing between large enterprises and SMEs. It is found that large enterprises exhibit a “U”-shaped effect in the dynamic evolution of data sharing, whereas SMEs tend to be more proactive. Beyond factors such as revenue, cost, and subsidies, data risk significantly influences the willingness of both enterprise types to share data. For digital platforms, service cost is the primary determinant in enabling data sharing among large enterprises and SMEs. The findings of this paper provide new insights and theoretical foundations for governmental policy formulation or enhancement, holding certain practical implications. For example, it enriches platform governance and other theories related to the platform economy, thereby contributing to its better development. Additionally, the government could formulate incentive policies tailored to the disparities between large enterprises and SMEs to foster data sharing and cooperative growth.

## Basic hypothesis and model construction of evolutionary game

### Problem description

In the development of global digital economy, data, as a key production factor, has become an important resource for the development of enterprises in the digital era. Drawing from the Resource-Based View^36^, the value of multiple data sets integrated and reorganized together is much greater than that of a single data set. Consequently, enterprises possessing more comprehensive data sets, with broader scope, can make more precise decisions based on data analytics, thereby mitigating the risk of information asymmetry to a certain extent^37^. This underscores the necessity for data sharing among enterprises^38^. However, in reality, disparities in resources and capabilities between large enterprises and SMEs, coupled with the absence of pertinent mechanisms and the inherent risks associated with data sharing, result in a lack of enthusiasm for data share between these entities.

**Figure 1:**
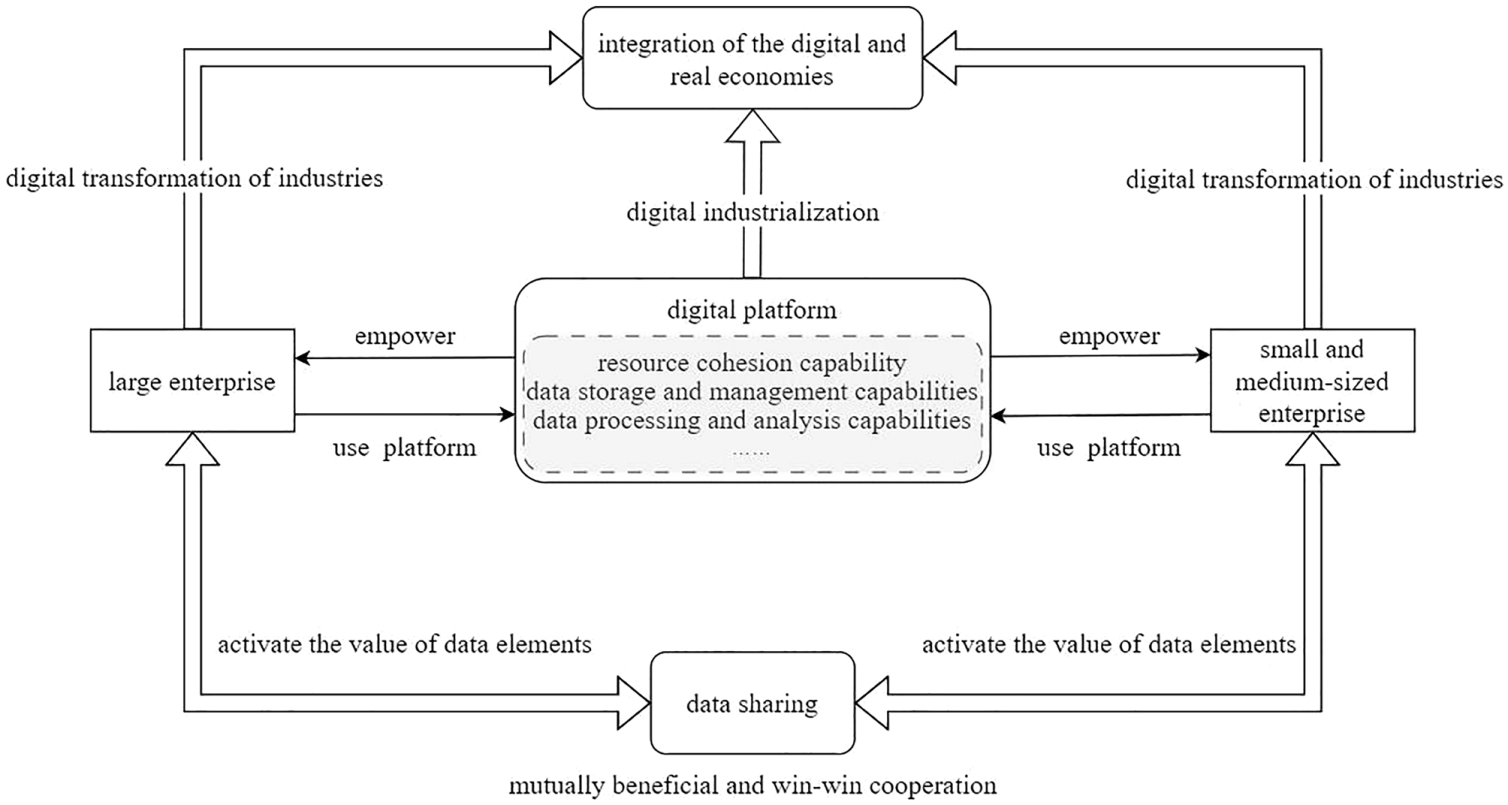
Data sharing of large, SMEs empowered by platform.

Considering that many countries around the world have begun to regard data as an important strategic resource, this paper attempts to build a new model of cooperative development in which digital platforms empower large enterprises and SMEs to share data and complement each other’s resources (shown in Fig. 1). Such cooperation is predicated on governmental policy incentives and the continuous improvement of the data security law.

In the digital era, digital platforms are not only the infrastructure to drive the digital transformation of enterprises, but also a powerful support tool for data sharing between large enterprises and SMEs. Although a few leading enterprises have built “derivative platforms”, such as China Haier’s COSMOPlat, the majority of large enterprises and SMEs are difficult to build mature digital platforms due to deficiencies in relevant technology, talent and other factors. Consequently, they often rely on third-party platforms for empowerment. Therefore, this paper chooses the third-party digital platform as the research object in the subsequent model.

With the empowerment of digital platforms, data sharing between large enterprises and SMEs can break the data silos between enterprises, fostering a more integrated cooperative ecosystem within the supply chain. This not only maximizes the potential value of data but also catalyzes industrial transformation and upgrading, achieving mutual benefits and win–win results. Specifically, large enterprises sharing data resources to SMEs, SMEs can obtain more resources and technical support, thus improving the capacity of digital transformation^39^, and activating the value of data elements empowered by digital platforms to reduce internal costs, increase efficiency, and expand external market growth^40^. Simultaneously, data sharing also promotes the transformation of large enterprises from hierarchical and empirical management to platform-based and data-driven management, thereby elevating quality and efficiency. In addition, platform empowerment is a process of value co-creation between digital and traditional enterprises^41^. The expansive user base of platforms correlates with an increased market scope and enhanced profitability^42^, which in turn bolsters the development of digital industry represented by digital platforms.

### Model assumptions

#### Hypothesis 1

According to evolutionary game theory^43^, the decision-making process of large enterprises, SMEs and digital platforms is a repeated game in which they constantly adjust and improve their strategic choices according to their own benefits under bounded rationality. The strategy choice of large enterprises and SMEs is (data sharing, no data sharing), and the probability of data sharing of large enterprises is x, the probability of no data sharing is (1 − x), 0 ≤ x ≤ 1; For SMEs, the probability of data sharing is y, and the probability of no data sharing is (1 − y), 0 ≤ y ≤ 1; The strategy choice of digital platform is (cooperation, non-cooperation), the probability of cooperation is z, the probability of non-cooperation is (1 − z), 0 ≤ z ≤ 1.

#### Hypothesis 2

According to the theory of resource dependence^44^, it is difficult for enterprises to obtain long-term benefits by relying only on their own resources. Sharing data with other businesses can address issues of information asymmetry and adverse selection, reduce communication and coordination costs^45^, and enable enterprises to combine their own characteristics to create unique resources. This approach can provide a competitive edge and enhance organizational efficiency^24^. In this process, enterprises need to conduct in-depth mining and analysis of massive raw data. In the digital era, they can utilize digital platforms that offer data mining, data analytics, machine learning, and other technological services to conduct comprehensive raw data analysis, thereby fully release the value of data^46^. Therefore, it is assumed that ${R}_{l}$ and ${R}_{ms}$ represent the initial returns of large enterprises and SMEs respectively; ${U}_{l}$ and ${U}_{ms}$ respectively represent the maximum benefits that large enterprises and SMEs can obtain from data sharing under the platform empowerment, and $\alpha$ represent the service quality of digital platforms, which will directly affect the benefits of enterprises in data sharing. Therefore, $\alpha {U}_{l}$ and ${\alpha U}_{ms}$ respectively represent the direct benefits that large enterprises and SMEs can obtain from data sharing. ${C}_{l}$ and ${C}_{ms}$ respectively represent the costs paid by large enterprises and SMEs to choose digital platform to be empowered. Because large enterprises have more internal organizations and more complicated business data, so they often need to customize services, and their digital inputs are larger than those of SMEs. If only large enterprises are willing to share data and cooperate with digital platform, then large enterprises can obtain platform services and share data with other large enterprises to gain benefits $\alpha {U}_{l}/2$, while SMEs will suffer losses ${K}_{ms}$ due to information asymmetry because they do not participate in data sharing; On the contrary, SMEs can gain $\alpha {U}_{ms}/2$, while large enterprises will lose ${K}_{l}$ due to information asymmetry. In the case of non-cooperation between digital platforms, enterprises will share data information through traditional methods such as telephone and email. In this case, large enterprises and SMEs need to pay higher human and material costs ${I}_{l}$ and ${I}_{ms}$ respectively to realize data resource sharing and obtain benefits ${V}_{l}$ and ${V}_{ms}$. If only large enterprises carry out data sharing, due to the reduction of the number of enterprises, the cost to be paid is also reduced to ${I}_{l}/2$, and the benefits are reduced to ${V}_{l}/2$; On the contrary, SMEs need to pay costs ${I}_{ms}/2$ and get benefits ${V}_{ms}/2$.

#### Hypothesis 3

According to the Market Failure Model, the market mechanism may sometimes fail to achieve the effective allocation of resources, necessitating government intervention to correct market failures^47^. For SMEs, they often struggle to secure sufficient resources for digital activities through the market mechanism due to constraints in capital, technology, and market access^48^. Although large enterprises have strong comprehensive strength, they are generally reluctant to share data with SMEs because doing so can increase their costs and potentially undermine their competitiveness. As the hub of data sharing, digital platforms may leverage their technological advantages to engage in monopolistic behaviors, which raises the threshold for enterprises to use the platform^49^. This, coupled with information asymmetry between enterprises and platforms with data security problems, which hinders enterprise data sharing. Therefore, government financial subsidies can help SMEs to obtain more resources, enhance the enthusiasm of large enterprises to share data, and incentivize digital platforms to lower barriers, providing secure and reliable data sharing services for both large enterprises and SMEs, thus fostering their cooperative development. It should be noted that the role of government intervention is limited, and its purpose is to help the market adjustment mechanism to return to normal, not to suppress market adjustment, so the government intervention should be moderate^50^. Therefore, it is assumed that in the case where the platform empowers data sharing among large and small enterprises, the government subsidies available to large enterprises, SMEs, and digital platforms are ${S}_{l}$, ${S}_{ms}$ and ${S}_{p}$, respectively.

#### Hypothesis 4

In the digital economy era, data has become an vital asset of enterprises and contain great commercial value. Data sharing implies the flow of data among multiple subjects. However, without comprehensive legal policies and adequate security measures, enterprises may face risks such as data leakage and data misuse. Although digital platforms serve as an ideal infrastructure for data sharing, are also potentially exposed to risks such as insecure transmission, system vulnerabilities, and cyberattacks. For example, Meta (formerly Facebook) experienced three data breaches in 2018, two of which were caused by system vulnerabilities. IBM’s statistics show that more than 25% of data breaches originated from human factors, and again from hacking, with another 48% of breaches resulting from hacking. In response to security issues in data sharing, many countries have begun to formulate relevant laws and policies to strengthen the protection of data security. In addition, the emergence of blockchain and other technologies has provided technical support for digital platforms to reduce data risks and safeguard data security^51^. Therefore, assuming that $\lambda$ is the degree of perfection of data security governance framework, the degree of perfection of data security guarantee framework will directly affect the security of data. When enterprises do not share data through digital platforms, the possible losses due to data privacy leakage for large enterprises and SMEs are $\left(1-\lambda \right){D}_{l}$ and $\left(1-\lambda \right){D}_{ms}$, respectively. whereas, when enterprises choose to cooperate with digital platforms, the technology of digital platforms can effectively reduce the risk of data sharing, so the possible losses due to data privacy leakage for large enterprises and SMEs are $\left(1-\lambda \right){D}_{l}/2$ and $\left(1-\lambda \right){D}_{ms}/$ 2, respectively. Where ${D}_{l}$ and ${D}_{ms}$ are the maximum possible loss of data privacy leakage of large enterprises and SMEs, respectively, and large enterprises tend to have rich data stocks involving more privacy secrets, and the losses arising from data privacy leakage are usually greater than those of SMEs. $Q$ represents the related cost that the digital platform needs to increase with the continuous improvement of the data security guarantee framework; when $\lambda \hspace{0.17em}$< 0.5, $Q\hspace{0.17em}$= 0; otherwise, when $\lambda \hspace{0.17em}$≥ 0.5, $Q$>0.

#### Hypothesis 5

According to the theory of Customer Relationship Management (CRM), enterprises can gain sustainable benefits by establishing long-term and intimate customer relationships^52^. In data sharing between large enterprises and SMEs, enterprises act as the platform’s customers, and the digital platform establishes a good cooperative relationship with them by providing high-quality products and services, thereby encouraging enterprise loyalty and reliance on the platform’s offerings. This dependency instills a willingness to perpetuate cooperation with the platform. Consequently, the platform accrues sticky benefits. Therefore, assuming that in the case that the digital platform enables large enterprises and SMEs, ${R}_{p}$ and ${C}_{p}$ respectively represent the platform’s direct revenue and service cost. $E$ represents the maximum sticky revenue that can be generated by the cooperation between the digital platform and enterprises, and the service quality of the platform will affect the dependence of enterprises on the platform, so $\alpha E$ represents the sticky revenue that can be obtained by the digital platform.

The meanings of parameters in Hypothesis are shown in Table 1.

**Table 1:** Model symbols and their meanings.

Parameters	Meaning	Parameters	Meaning
$\alpha$	Digital platform service quality,$\alpha \in \left[\mathrm{0,1}\right]$	${V}_{ms}$	The direct benefits of traditional data sharing for SMEs
$\lambda$	Degree of completeness of data security guarantee framework,$\lambda \in \left[\mathrm{0,1}\right]$	${C}_{ms}$	And the input cost of data sharing through the platform for SMEs
${R}_{l}$	Initial benefits for large enterprises	${I}_{ms}$	Costs for SMEs to share data resources through traditional means
${U}_{l}$	The maximum benefits that can be obtained from data sharing in large enterprises under platform empowerment	${D}_{ms}$	The largest possible loss of data privacy leakage in SMEs
${V}_{l}$	The direct benefits that can be obtained from data sharing in large enterprises	${S}_{ms}$	The amount of government subsidies for digital transformation of SMEs
${C}_{l}$	Input costs for large enterprises to share data through the platform	${K}_{ms}$	Losses arising from information asymmetry in SMEs
${I}_{l}$	The cost of traditional data resource sharing for large enterprises	$y$	Probability of digital transformation for SMEs,$y\in \left[\mathrm{0,1}\right]$
${D}_{l}$	And the largest possible loss caused by data privacy leakage in large enterprises	${R}_{p}$	The direct benefits obtained by the platform from cooperation with large enterprises and SMEs
${S}_{l}$	The amount of government subsidies to large enterprises for active traction	$E$	Sticky revenue of the platform
${K}_{l}$	The loss of large enterprises due to information asymmetry	${C}_{p}$	The operating costs of cooperation between the platform and large enterprises and SMEs
$x$	The probability that large enterprises choose active traction,$x\in \left[\mathrm{0,1}\right]$	${S}_{p}$	And the amount of government subsidies for the cooperation between the platform and large enterprises and SMEs
${R}_{ms}$	Initial income of SMEs	$Q$	Increased costs related to the platform due to the improvement of the data governance framework,
${U}_{ms}$	And the maximum benefits that can be obtained from data sharing of SMEs under platform empowerment	$z$	The probability that the platform chooses “cooperation”,$z\in \left[\mathrm{0,1}\right]$

### Model construction

According to the above assumptions and different strategy choices of large enterprises, SMEs and digital platforms, the payoff matrix of the three-party mixed strategy game can be constructed (as shown in Table 2).

**Table 2:** Three-party game matrix.

Large enterprises	SMEs	Digital platforms	
		Cooperative (z)	Uncooperative (1 − z)
Sharing (x)	Sharing (y)	${R}_{l}+\alpha {U}_{l}+{S}_{l}-{C}_{l}-\left(1-\lambda \right){D}_{l}/2$ ${R}_{ms}+\alpha {U}_{ms}+{S}_{ms}-{C}_{ms}-(1-\lambda ){D}_{ms}/2$ ${R}_{p}+{S}_{p}+\alpha E-{C}_{p}-Q$	${R}_{l}+{V}_{l}-{I}_{l}-\left(1-\lambda \right){D}_{l}$ ${R}_{ms}+{V}_{ms}-{I}_{ms}-(1-\lambda ){D}_{ms}$ $0$
	Not sharing (1 − y)	${R}_{l}+\alpha {U}_{l}/2+{S}_{l}-{C}_{l}-\left(1-\lambda \right){D}_{l}/2$ ${R}_{ms}-{K}_{ms}$ ${R}_{p}/2+{S}_{p}/2+\alpha E/2-{C}_{p}/2-Q$	${R}_{l}+{V}_{l}/2-{I}_{l}/2-\left(1-\lambda \right){D}_{l}$ ${R}_{ms}-{K}_{ms}$ $0$
Not sharing (1 − x)	Sharing (y)	${R}_{l}-{K}_{l}$ ${R}_{ms}+\alpha {U}_{ms}/2+{S}_{ms}-{C}_{ms}-(1-\lambda ){D}_{ms}/2$ ${R}_{p}/2+{S}_{p}/2+\alpha E/2-{C}_{p}/2-Q$	${R}_{l}-{K}_{l}$ ${R}_{ms}+{V}_{ms}/2-{I}_{ms}/2-(1-\lambda ){D}_{ms}$ $0$
	Not sharing (1 − y)	${R}_{l}$ ${R}_{ms}$ $0$	${R}_{l}$ ${R}_{ms}$ $0$

## Evolutionary game replication dynamic equation and stability analysis

### Replication dynamic equation

According to the stability theorem of dynamic differential equations, the replication dynamic equations of the players of the three-party game are established respectively. Let the expected revenue of A large enterprise choosing the strategy of “data sharing” and “no data sharing” be A_x1_ and A_x2_ respectively, and the average expected revenue is A_x3_, then:

$$\left\{ \begin{aligned} A_{x1} & = yz\left[ {R_{l} + \alpha U_{l} + S_{l} - C_{l} - \frac{{(1 - \lambda )D_{l} }}{2}} \right] \\ & \quad + y(1 - z)(R_{l} + V_{l} - I_{l} - (1 - \lambda )D_{l} ) \\ & \quad + z(1 - y)\left[ {R_{l} + \alpha \frac{{U_{l} }}{2} + S_{l} - C_{l} - \frac{{(1 - \lambda )D_{l} }}{2}} \right] \\ & \quad + (1 - y)(1 - z)\left( {R_{l} + \frac{{V_{l} }}{2} - \frac{{I_{l} }}{2} - (1 - \lambda )D_{l} } \right) \\ A_{x2} & = yz(R_{l} - K_{l} ) + y(1 - z)(R_{l} - K_{l} ) + z(1 - y)R_{l} + (1 - y)(1 - z)R_{l} \\ A_{x3} & = xA_{x1} + (1 - x)A_{x2} \\ \end{aligned} \right.$$

The replication dynamic equation is:

$$\begin{aligned} F(x) & = \frac{dx}{{dt}} = x(1 - x)(A_{x1} - A_{x2} ) \\ = x(1 - x)\left[ {yz\alpha \frac{{U_{l} }}{2} + yK_{l} - \left( {1 - \frac{z}{2}} \right)(1 - \lambda )D_{l} + z\left( {\alpha \frac{{U_{l} }}{2} + S_{l} - C_{l} } \right) + (1 + y)(1 - z)\left( {\frac{{V_{l} }}{2} - \frac{{I_{l} }}{2}} \right)} \right] \\ \end{aligned}$$

Similarly, if the expected revenue of SMEs choosing “data sharing” or “no data sharing” strategy is A_y1_ and A_y2_, and the average expected revenue is A_y3_, then:

$$\left\{ \begin{aligned} A_{y1} & = xz\left[ {R_{ms} + \alpha U_{ms} + S_{ms} - C_{ms} - \frac{{(1 - \lambda )D_{ms} }}{2}} \right] \\ & \quad + x(1 - z)(R_{ms} + V_{ms} - I_{ms} - (1 - \lambda )D_{ms} ) \\ & \quad + z(1 - x)\left[ {R_{ms} + \alpha \frac{{U_{ms} }}{2} + S_{ms} - C_{ms} - \frac{{(1 - \lambda )D_{ms} }}{2}} \right] \\ & \quad + (1 - x)(1 - z)\left( {R_{ms} + \frac{{V_{ms} }}{2} - \frac{{I_{ms} }}{2} - (1 - \lambda )D_{ms} } \right) \\ A_{y2} & = xz(R_{ms} - K_{ms} ) + x(1 - z)R_{ms} + z(1 - x)R_{ms} + (1 - x)(1 - z)R_{ms} \\ A_{y3} & = yA_{y1} + (1 - y)A_{y2} \\ \end{aligned} \right.$$

The replication dynamic equation is as follows:

$$\begin{aligned} F(y) & = \frac{dy}{{dt}} = y(1 - y)(A_{y1} - A_{y2} ) \\ & \quad = y(1 - y)\left[ {xz\alpha \frac{{U_{ms} }}{2} + xK_{ms} - \left( {1 - \frac{z}{2}} \right)(1 - \lambda )D_{ms} } \right. \\ & \quad \left. { + z\left( {a\frac{{U_{ms} }}{2} + S_{ms} - C_{ms} } \right) + (1 + x)(1 - z)\left( {\frac{{V_{ms} }}{2} - \frac{{I_{ms} }}{2}} \right)} \right] \\ \end{aligned}$$

Similarly, suppose that the expected returns of the digital platform choosing “cooperation” and “non-cooperation” strategies are A_z1_ and A_z2_, and the average expected returns are A_z3_, then:

$$\left\{ \begin{aligned} A_{z1} & = xy(R_{p2} + S_{p} + \alpha E - C_{p} - Q) + x(1 - y)\left( {\frac{{R_{p} }}{2} + \frac{{S_{p} }}{2} + \frac{\alpha E}{2} - \frac{{C_{p} }}{2} - Q} \right) \\ & \quad + y(1 - x)\left( {\frac{{R_{p} }}{2} + \frac{{S_{p} }}{2} + \frac{\alpha E}{2} - \frac{{C_{p} }}{2} - Q} \right) + (1 - x)(1 - y)0 \\ A_{z2} & = xy0 + x(1 - y)0 + y(1 - x)0 + (1 - x)(1 - y)0 \\ A_{z3} & = zA_{z1} + (1 - z)A_{z2} \\ \end{aligned} \right.$$

The replication dynamic equation is as follows:

$$\begin{aligned} F(z) & = \frac{dz}{{dt}} = z(1 - z)(A_{z1} - A_{z2} ) \\& = z(1 - z)\left[ {xyQ + (x + y)\left( {\frac{{R_{p} }}{2} + \frac{{S_{p} }}{2} + \frac{\alpha E}{2} - \frac{{C_{p} }}{2} - Q} \right)} \right] \\ \end{aligned}$$

### Stability analysis of three-party evolution strategy

The players of the evolutionary game can realize the stability strategy of the system under the joint action. Since mixed strategies are not evolutionarily stable in asymmetric games^53^, only the evolutionary stability of pure strategies is discussed. Set ${\text{F}}\left({\text{x}}\right)={\text{F}}\left({\text{y}}\right)={\text{F}}\left({\text{z}}\right)=0$. The eight pure strategy equilibria of “large enterprise”, “SMEs” and “digital platform” in the process of game can be obtained E_1_ (0,0,0), E_2_ (0,0,1), E_3_ (0,1,0), E_4_ (0,1,1), E_5_ (1,0,0), E_6_ (1,0,1), E_7_ (1,1,0), E_8_ (1,1,1). According to the Lyapunov criterion, when all the eigenvalues of the Jacobi matrix are less than 0^30^, the equilibrium point is the evolutionary stable point of the system, and the Jacobi matrix is as follows:

$$J=\left[\begin{array}{ccc}\partial F(x)/\partial x& \partial F(x)/\partial y& \partial F(x)/\partial z\\ \partial F(y)/\partial x& \partial F(y)/\partial y& \partial F(y)/\partial z\\ \partial F(z)/\partial x& \partial F(z)/\partial y& \partial F(z)/\partial z\end{array}\right]$$

The eigenvalues of the Jacobi matrix corresponding to the above eight pure strategy equilibrium points are shown in Table 3.

**Table 3:** Eigenvalues and stability of the matrix corresponding to the equilibrium points.

Point of equilibrium	Eigenvalues ${\lambda }_{1}$	Eigenvalues ${\lambda }_{2}$	Eigenvalues ${\lambda }_{3}$
E_1_ (0,0,0)	$\frac{{V}_{l}}{2}-\frac{{I}_{l}}{2}-\left(1-\lambda \right){D}_{l}$	$\frac{{V}_{ms}}{2}-\frac{{I}_{ms}}{2}-(1-\lambda ){D}_{ms}$	0
E_2_ (0,0,1)	$\alpha \frac{{U}_{l}}{2}+{S}_{l}-{C}_{l}-\left(1-\lambda \right){D}_{l}$	$\alpha \frac{{U}_{ms}}{2}+{S}_{ms}-{C}_{ms}-(1-\lambda ){D}_{ms}$	0
E_3_ (0,1,0)	${K}_{l}{+V}_{l}-{I}_{l}-\left(1-\lambda \right){D}_{l}$	$\frac{{I}_{ms}}{2}-\frac{{V}_{ms}}{2}+(1-\lambda ){D}_{ms}$	$\frac{{R}_{p}}{2}+\frac{{S}_{p}}{2}+\frac{\alpha E}{2}-\frac{{C}_{p}}{2}-Q$
E_4_ (0,1,1)	$\alpha {U}_{l}+{K}_{l}+{S}_{l}-{C}_{l}-\left(1-\lambda \right){D}_{l}$	$-\alpha \frac{{U}_{ms}}{2}-{S}_{ms}+{C}_{ms}+(1-\lambda ){D}_{ms}$	$-\frac{{R}_{p}}{2}-\frac{{S}_{p}}{2}-\frac{\alpha E}{2}+\frac{{C}_{p}}{2}+Q$
E_5_ (1,0,0)	$\frac{{I}_{l}}{2}-\frac{{V}_{l}}{2}+\left(1-\lambda \right){D}_{l}$	${K}_{ms}+{V}_{ms}-{I}_{ms}-\left(1-\lambda \right){D}_{ms}$	$\frac{{R}_{p}}{2}+\frac{{S}_{p}}{2}+\frac{\alpha E}{2}-\frac{{C}_{p}}{2}-Q$
E_6_ (1,0,1)	$-\alpha \frac{{U}_{l}}{2}-{S}_{l}+{C}_{l}+\left(1-\lambda \right){D}_{l}$	$\alpha {U}_{ms}+{S}_{ms}+{K}_{ms}-{C}_{ms}-\left(1-\lambda \right){D}_{ms}$	$-\frac{{R}_{p}}{2}-\frac{{S}_{p}}{2}-\frac{\alpha E}{2}+\frac{{C}_{p}}{2}+Q$
E_7_ (1,1,0)	$-{K}_{l}{-V}_{l}+{I}_{l}+\left(1-\lambda \right){D}_{l}$	$-{K}_{ms}-{V}_{ms}+{I}_{ms}+(1-\lambda ){D}_{ms}$	${R}_{p}+{S}_{p}+\alpha E-{C}_{p}-Q$
E_8_ (1,1,1)	$-\alpha {U}_{l}-{S}_{l}-{K}_{l}+{C}_{l}+\left(1-\lambda \right){D}_{l}$	$-\alpha {U}_{ms}-{S}_{ms}-{K}_{ms}+{C}_{ms}+\left(1-\lambda \right){D}_{ms}$	$-{R}_{p}-{S}_{p}-\alpha E+{C}_{p}+Q$

According to the eigenvalues of Jacobi matrix, the equilibrium points E_1_ (0,0,0) and E_2_ (0,0,1) have eigenvalues of 0, so they are unstable points; The stability of the remaining six equilibrium points can be discussed in the following six situations.

*Case* 1: when ${V}_{l}-{I}_{l}-\left(1-\lambda \right){D}_{l}$<$-{K}_{l}$,${V}_{ms}/2-{I}_{ms}/2-\left(1-\lambda \right){D}_{ms}$>$0$,${ R}_{p}/2+{S}_{p}/2+\alpha E/2-{C}_{p}/2-Q$<0, only E_3_(0,1,0) is an evolutionarily stable point. In this case, the digital platform chooses not to cooperate because it cannot benefit from enabling. In this case, large enterprises will choose not to share data resources due to the high cost of cooperation with SMEs, but SMEs can still benefit from traditional resource sharing, so SMEs will cooperate with each other.

*Case* 2: when $\alpha {U}_{l}+{S}_{l}-{C}_{l}-\left(1-\lambda \right){D}_{l}$<$-{K}_{l}$,$\alpha {U}_{ms}/2+{S}_{ms}-{C}_{ms}-(1-\lambda ){D}_{ms}$>$0$,${ R}_{p}/2+{S}_{p}/2+\alpha E/2-{C}_{p}/2-Q$>0, only E_4_(0,1,1) is the evolutionary equilibrium point. In this case, if large enterprises share data with SMEs through platform empowerment, they will incur great loss, so they will not choose to share data with SMEs. In the case that the platform only enables SMEs, both the platform and SMEs can benefit, and both parties will choose the cooperation strategy.

*Case* 3: when ${V}_{l}/2-{I}_{l}/2-\left(1-\lambda \right){D}_{l}$>0,${V}_{ms}-{I}_{ms}-\left(1-\lambda \right){D}_{ms}$>$0$, ${R}_{p}/2+{S}_{p}/2+\alpha E/2-{C}_{p}/2-Q$<0, only E_5_(1,0,0) is the evolutionary equilibrium point. In this case, the digital platform chooses not to cooperate because it cannot benefit from enabling. In this case, SMEs will choose not to share data resources due to the high cost of cooperation with large enterprises. However, large enterprises can still benefit from traditional resource sharing, so large enterprises will cooperate with each other.

*Case* 4: when $\alpha {U}_{l}/2+{S}_{l}-{C}_{l}-\left(1-\lambda \right){D}_{l}$>$0$,$\alpha {U}_{ms}+{S}_{ms}-{C}_{ms}-\left(1-\lambda \right){D}_{ms}$<$-{K}_{ms}$,${ R}_{p}/2+{S}_{p}/2+\alpha E/2-{C}_{p}/2-Q$>0, only E_6_(1,0,1) is the evolutionary equilibrium point. In this case, if SMEs share data with large enterprises through platform empowerment, there will be a large loss, so they will not choose to share data with large enterprises. In the case that the platform only enables large enterprises, both the platform and large enterprises can benefit, and both parties will choose the cooperation strategy.

*Case* 5: when ${K}_{l}{+V}_{l}-{I}_{l}-\left(1-\lambda \right){D}_{l}$>0,${K}_{ms}+{V}_{ms}-{I}_{ms}-\left(1-\lambda \right){D}_{ms}$>0,${ R}_{p}+{S}_{p}+\alpha E-{C}_{p}-Q$<0, only E_7_(1,1,0) is the evolutionary equilibrium point. In this case, the platform will choose not to participate in the cooperation because it cannot benefit from enabling large enterprises and SMEs to share data. However, large enterprises and SMEs can still benefit from the traditional resource sharing mode, and the two sides will carry out data resource sharing cooperation.

*Case* 6: when $\alpha {U}_{l}+{S}_{l}-{C}_{l}-\left(1-\lambda \right){D}_{l}$>${K}_{l}$,$\alpha {U}_{ms}+{S}_{ms}-{C}_{ms}-\left(1-\lambda \right){D}_{ms}$>${K}_{ms}$,${R}_{p}+{S}_{p}+\alpha E-{C}_{p}-Q$>0, only E_8_(1,1,1) is the evolutionary equilibrium point. In this case, when the digital platform enables large enterprises and SMEs to share data, both the platform and the enterprises can benefit, and finally form the ideal state of tripartite cooperation.

## Numerical analysis of evolutionary game

To enhance the model simulation results’ proximity to the actual situation, this study augments the interpretability and reliability of the results by parameterizing them with reference to actual cases and pertinent policies. Specifically, we analyze cases from China’s “*Case Collection of Typical Models of Integration and Innovation of Large, Small and Medium-sized Enterprises*” are selected to be analyzed. Among them, Zoomlion Heavy Industries Co., Ltd. leverages a platform to drive cloud and intelligent operations for SMEs within the industrial chain, fostering data integration among large enterprises and SMEs. This integration helps SMEs to achieve 30% increase in scale efficiency, 10% reduction in production cost, and 10% improvement in enterprise cooperative quality, thereby extending the overall value chain of the construction machinery. The COSMOPlat, recognizing the diverse needs of SMEs at various stages of transformation, offers flexible and cost-effective solutions. It also provides customized digital services to large enterprises, propelling the integrated innovation and development of large enterprises and SMEs within the industrial chain. In the process of helping Tianhui Dairy’s transformation, reduce equipment maintenance cost by 40%, improve comprehensive efficiency by 20%, spare parts inventory by 20%^54^.

At present, more and more digital platforms are retaining customers through subscription services, thereby capturing a larger market share and generating sticky revenue. In the “*2022 Annual Performance Report of Kingdee*”^55^, the annual recurring revenue of Kingdee cloud subscription service accounts for approximately 60% of the total cloud business revenue. Enterprises have different digital investment according to their own strength and scale. According to “*the digital transformation report of Dongguan City*”^56^, the digital investment of enterprises in the next three years is mainly between 1 to 3 million yuan and 3 to 5 million yuan. Regarding government incentive policy, refer to the digital subsidy policy of enterprises in various parts of China, where the subsidy typically ranges from 20 to 30% of the cost. Furthermore, according to the national policy^57^, the subsidy to the platform does not exceed 30% of the cost. Concurrently, in the “*2022 Data Leakage Cost Report*” released by IBM^58^, it is mentioned that the secondary data leakage rate is close to 50%, causing substantial direct and potential losses to enterprises.

Based on the ideal evolutionary stable point E_8_(1,1,1), combined with the above cases, reports and relevant policies, referring to the division method of large enterprises and SMEs in China^59^, considering the differences in value creation ability^60^, risk bearing ability and other aspects among different subjects, and referring to the idea of parameter assignment by Wei et al.^34^ and Li et al.^61^, setting parameters $\alpha \hspace{0.17em}$= 0.7, $\lambda \hspace{0.17em}$= 0.5, ${R}_{l}\hspace{0.17em}$= 20, ${R}_{ms}\hspace{0.17em}$= 10, ${R}_{p}\hspace{0.17em}$= 4.5, ${U}_{l}\hspace{0.17em}$= 10, ${U}_{ms}\hspace{0.17em}$= 6, ${V}_{l}\hspace{0.17em}$= 5, ${V}_{ms}\hspace{0.17em}$= 4, ${C}_{l}\hspace{0.17em}$= 2.5, ${C}_{ms}\hspace{0.17em}$= 2, ${C}_{p}\hspace{0.17em}$= 2.5, ${S}_{l}\hspace{0.17em}$= 0.65, ${S}_{ms}\hspace{0.17em}$= 0.6, ${S}_{p}\hspace{0.17em}$= 0.75, ${I}_{l}\hspace{0.17em}$= 3, ${I}_{ms}\hspace{0.17em}$= 2.5, ${D}_{l}\hspace{0.17em}$= 7, ${D}_{ms}\hspace{0.17em}$= 5, ${K}_{l}=1$, ${K}_{ms}$=2, $E$=3, $Q$=0.5. At the same time, the initial strategy value of the players in the three-party game is set as 0.5, and the evolution path of the players’ strategy selection is numerically analyzed by MATLAB software^62^.

### Numerical analysis of three-party evolutionary strategies

**Figure 2:**
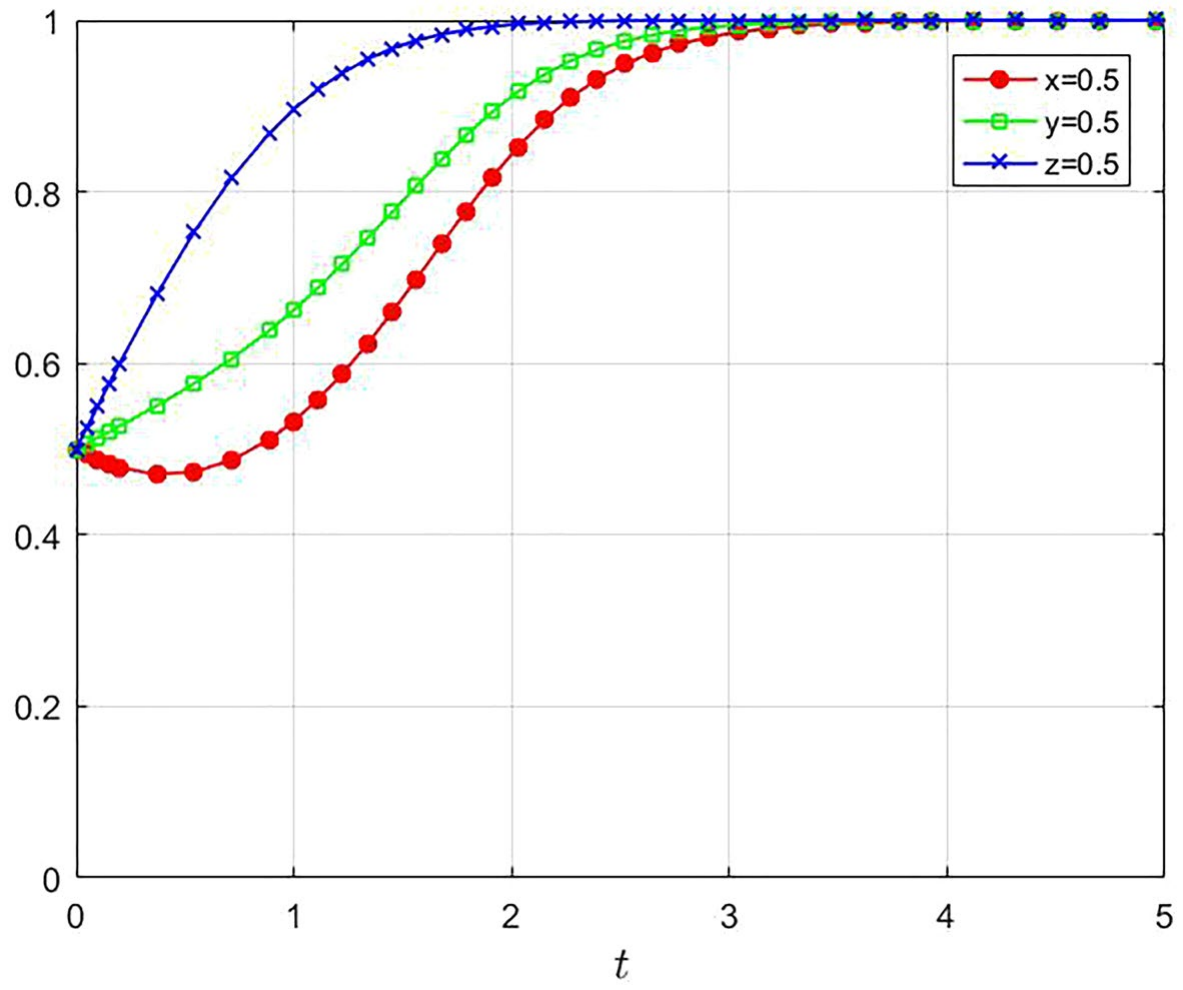
Numerical analysis of the three-party evolutionary game1.

Through the simulation analysis of the above values, the results are shown in Fig. 2. Under the constraints in Case 6, the main body of the tripartite evolutionary game will eventually evolve to the ideal equilibrium state E_8_(1,1,1). In this case, the tripartite strategy is to share data for large enterprises and SMEs, and the digital platform chooses to cooperate with the enterprises.

In addition, Fig. 2 shows that both digital platforms (z) and SMEs (y) demonstrate an increasing trend towards a stable state, with digital platforms evolve to stability in the fastest. For digital platforms, “empowerment” is an important mechanism to promote value co-creation and platform development. Cooperating with enterprises and providing services for them is the primary way for digital platform to gain revenue. Therefore, platforms will increase their willingness to cooperate and have a strong incentive to promote data sharing among enterprises. For SMEs, although data sharing requires higher costs, they can obtain more market information and resource support through data sharing with large enterprises, thus reducing information asymmetry, decreasing production and operation costs, increasing market share and bolstering competitiveness. Therefore, the willingness of SMEs to share data will also gradually increase.

However, it can be found that the evolution path of large enterprises (x) is U-shaped curve, with their data sharing willingness initially declining before ascending. This may be attributed to, on the one hand, large enterprises may incur large losses due to data privacy leakage during data sharing. Therefore, in a short period of time, large enterprises may prefer to protect their own data because of risk considerations, and the motivation for data sharing will decrease. On the other hand, large enterprises face competitive pressures from SMEs. If large enterprises share their data, it may lead to an increase in the competitiveness of SMEs, indirectly imperiling the market position and interests of large enterprises. As a result, large enterprises may adopt conservative strategies and reduce their willingness to share data in the short term.

And as the evolution time goes on, the sharing enthusiasm of digital platforms and SMEs gradually increases. And large enterprises may find after comprehensive deliberation that platform empowerment and data sharing can bring them more benefits, such as accessing new business opportunities brought by external data, enhancing efficiency, and reducing costs. As a result, the probability of large enterprises’ data sharing gradually rises. As the game deepens, all parties gradually see the possibility of win–win cooperation, thereby augmenting the willingness to cooperate. Ultimately, the ideal situation of digital platform empowering data sharing among large enterprises and SMEs is realized.

### Influence of the change of the initial strategy value of the platform on the strategy selection of enterprises

**Figure 3:**
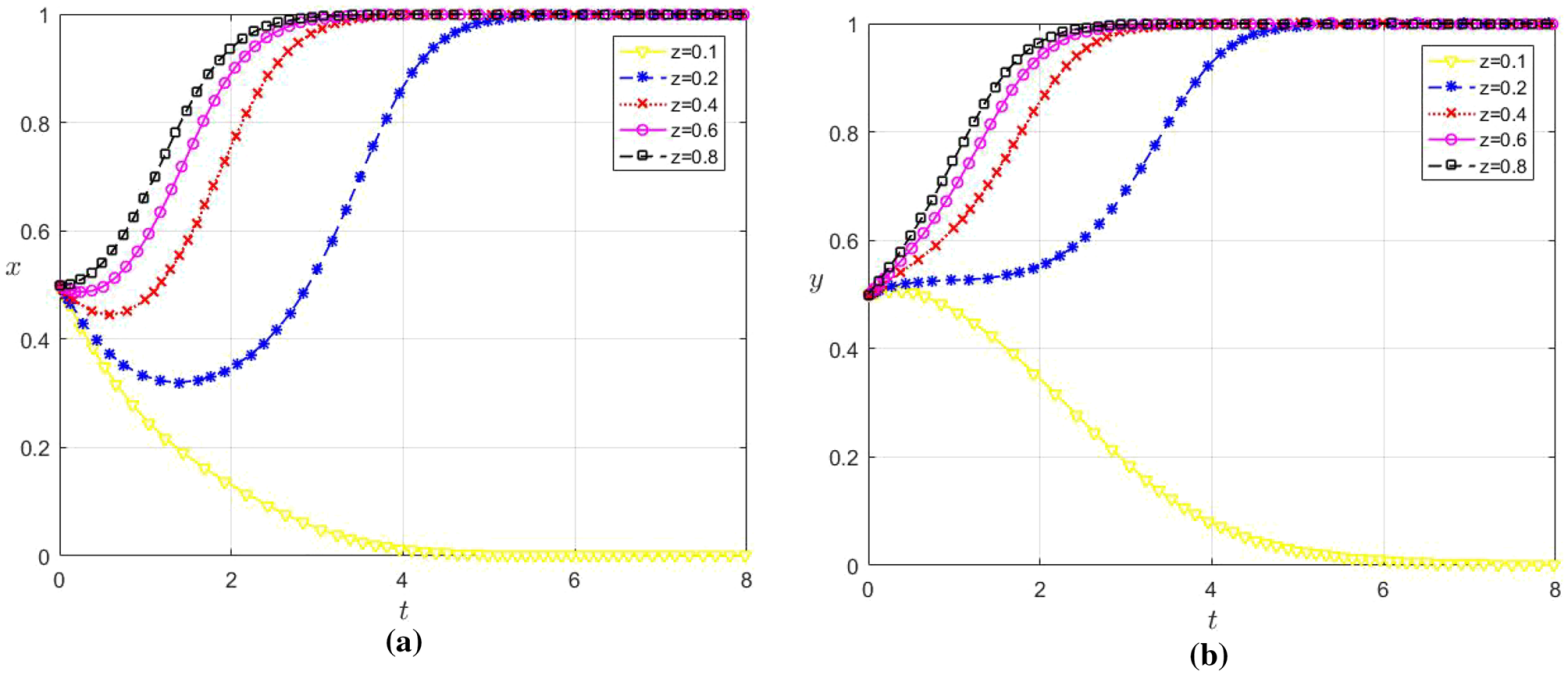
(**a**) The influence of z change on the strategy choice of large enterprises (**b**) the influence of z change on the strategy choice of SMEs2.

In order to analyze the influence of the change of the initial strategy value of the digital platform on the strategy choice of the enterprise, the values of z are set as 0.1, 0.2, 0.4, 0.6 and 0.8 respectively, and the other parameters remain unchanged. The evolution results are shown in Fig. 3a and b.

According to Fig. 3a and b, when z ≥ 0.2, both x and y converge to 1, with y converging faster than x. Conversely, when z ≤ 0.1, both x and y converge to 0. This suggests that there is a specific threshold above which the probability of cooperation with digital platforms becomes sufficiently high to induce enterprises to opt for data sharing. This reflects that at lower probabilities of cooperation, enterprises consider it challenging to maximize the value of data through conventional sharing methods. Coupled with inadequate security technology, the risk of data privacy breaches is elevated, potentially leading to more detriment than benefit for the enterprise. Consequently, when enthusiasm for digital platform cooperation wanes, risk aversion dictates that enterprises abstain from data sharing. And large enterprises will choose not to share data more quickly due to the greater potential loss.

In addition, Fig. 3a indicates that as a platform’s willingness to cooperate increases, the U-shaped effect observed in the evolution of large enterprises’ sharing strategies diminishes. This could be attributed to the digital platforms can reduce the difficulty and risk of data sharing by providing technical support and services to better create value for enterprises. Therefore, large enterprises will increase the data sharing motivation, thus reducing the U-shaped effect. Meanwhile, from Fig. 3b, it can be found that the willingness to cooperate with digital platforms correlates positively with SMEs’ data sharing motivation, which exceeds that of large enterprises. Given SMEs’ robust demand for data sharing, an augmented cooperative disposition from the platform is likely to draw more SMEs to participate. Therefore, influenced positively by the empowerment of SMEs via the platform, large enterprises may enhance their data sharing inclination, thus reducing the U-shaped effect.

### The influence of subsidies and costs on the selection of evolutionary game strategies

**Figure 4:**
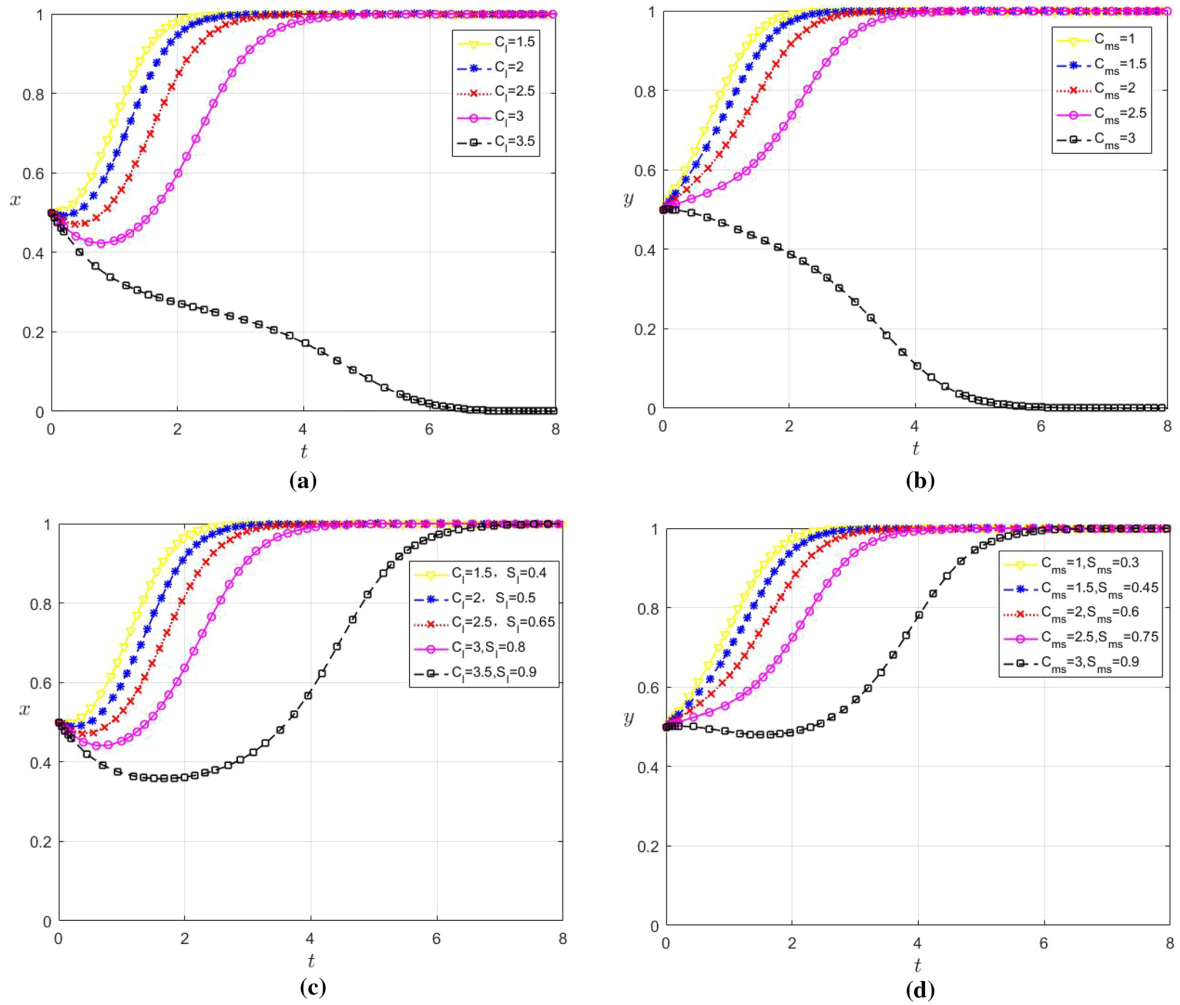
(**a**) Influence of ${C}_{l}$ change on the strategy choice of large enterprises (**b**) Influence of ${C}_{ms}$ change on the strategy choice of SMEs c Influence of ${C}_{l}$ and ${S}_{l}$ change on the strategy choice of large enterprises d Influence of ${C}_{ms}$ and ${S}_{ms}$ change on the strategy choice of SMEs.

**Figure 5:**
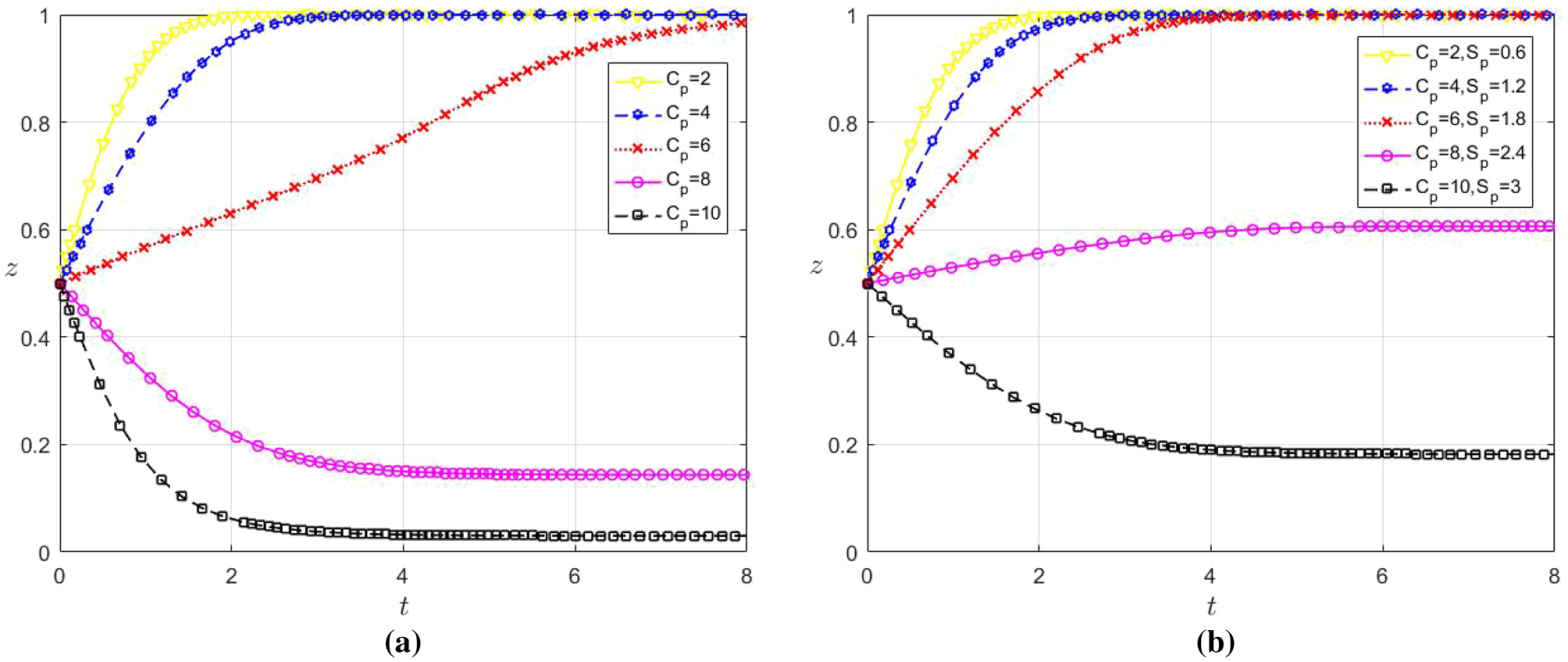
(**a**) Influence of ${C}_{p}$ change on the strategy choice of platform (**b**) Influence of ${C}_{p}$ and ${S}_{p}$ change on the strategy choice of platform.

1. The influence of data sharing cost on the strategy choice of the firm when the subsidy remains unchanged
  Under the condition that the government subsidy remains unchanged ${C}_{l}$, in order to analyze the influence of the change of the input cost of data sharing through platform empowerment on the strategy choice of large enterprises and SMEs, we set ${C}_{l}$ as 1.5, 2, 2.5, 3 and 3.5 respectively; Set ${C}_{ms}$ be 1, 1.5, 2, 2.5, 3, and the remaining parameter values remain unchanged. The evolution results are shown in Fig. 4a and b.
  Observations from Fig. 4a and b indicate that when ${C}_{l}\hspace{0.17em}$≤ 3 and ${C}_{ms}\hspace{0.17em}$≤ 2.5, both x and y converge to 1, and the convergence speed of y is faster than that of x. Conversely, when ${C}_{l}$ ≥ 3.5 and ${C}_{ms}$ ≥ 3, both x and y converge to 0. data sharing becomes a preferred option for large enterprises and SMEs when the associated costs are below a certain threshold. This reflects that low data sharing costs enable both enterprise types to derive benefits sufficient enough to offset their costs. At the same time, the cooperative relationship helps both parties to better recognize the long-term value and potential benefits of data sharing. Therefore, as the cost of data sharing decreases, the more motivated SMEs and large enterprises will engage in data sharing. However, when the cost for data sharing is high, large enterprises and SMEs will reassess the benefits and costs of data sharing, potentially leading to a decision against it if the costs outweigh the benefits.
  In addition, comparing Fig. 4a and b reveals that SMEs will choose not to data share more quickly than large enterprises when the cost is too high. This may be due to the fact that SMEs usually have relatively low risk tolerance. When faced with costly data sharing, they may be more inclined to adopt a conservative strategy to avoid taking excessive risks. As a result, SMEs will be quicker to opt out of data sharing.
2. The impact of subsidies and data sharing costs on the strategy choice of enterprises
  If the government subsidy is adjusted with the change of enterprise data sharing cost, in the case of (1) ${C}_{l}$ and ${C}_{ms}$ change, according to the government subsidy range is 20–30% of enterprise digital cost, let ${S}_{l}$ be 0.4, 0.5, 0.65, 0.8, 0.9 respectively; Let ${S}_{ms}$ be 0.3, 0.45, 0.6, 0.75 and 0.9 respectively, and the remaining parameters remain unchanged. The evolution results are shown in Fig. 4c and d.
  It can be seen from Fig. 4c and d that both x and y converge to 1. This reflects the efficacy of a flexible subsidy policy as a positive incentive, which can effectively compensate for the cost of data sharing among different enterprises and boost them participate in data sharing. In addition, government subsidy is also a signaling effect. It conveys to the market the government’s attitude of encouraging and supporting data sharing, which helps to enhance the willingness to cooperate and trust among enterprises. Therefore, a flexible subsidy policy can be adjusted according to the specific situation of different enterprises, thereby facilitating the realization of data sharing between large enterprises and SMEs.
3. The impact of digital platform service cost on the choice of platform strategy when the subsidy remains unchanged
  When the government subsidy remains unchanged, in order to analyze the influence of the change of digital platform service cost on the strategy choice of the game players, set ${C}_{p}$ as 2, 4, 6, 8 and 10 respectively, and the remaining parameters remain unchanged. The evolution results are shown in Fig. 5a.
  It can be seen from Fig. 5a that when ${C}_{p}$ ≤ 6, z converges to 1, and the ${C}_{p}$ smaller it is, the faster the convergence speed is. When ${C}_{p}$ ≥ 8, z decreases continuously as it increases. Therefore, there is a threshold at which digital platforms will choose not to cooperate with large enterprises and SMEs when the cost of the services greater than the threshold. This reflects that while empowering enterprises is an important way for platforms to gain revenue, when the cost of services exceed the gains may lead them to deem such partnerships economically impractical. As a result, digital platforms may focus their resources on more profitable areas and avoid high-cost data-sharing service cooperations with large enterprises and SMEs.
4. The impact of subsidies and digital platform service costs on platform strategy selection
  If the government subsidy is adjusted with the change of enterprise data sharing cost, in ${C}_{p}$ the case of change in (3), according to the government subsidy is 30% of the digital platform cost ${S}_{p}$, let them be 0.6, 1.2, 1.8, 2.4 and 3 respectively, and the remaining parameter values remain unchanged. The evolution results are shown in Fig. 5b.
  It can be seen from Fig. 5b that the government can alleviate the pressure on the operating costs of platforms by enhancing the subsidies, thereby stimulating platforms to empower enterprises. However, when the costs that the platform needs to pay is too high, but government subsidies are limited. If the revenue generated from digital platform-enterprise cooperations fails to offset their costs, platforms may still be disinclined to engage in cooperation. At this time, although government subsidies can improve the enthusiasm of platform cooperation to a certain extent, they cannot achieve a stable state of cooperation.
5. The impact of government subsidies on the selection of evolutionary game strategies when the cost is constant

**Figure 6:**
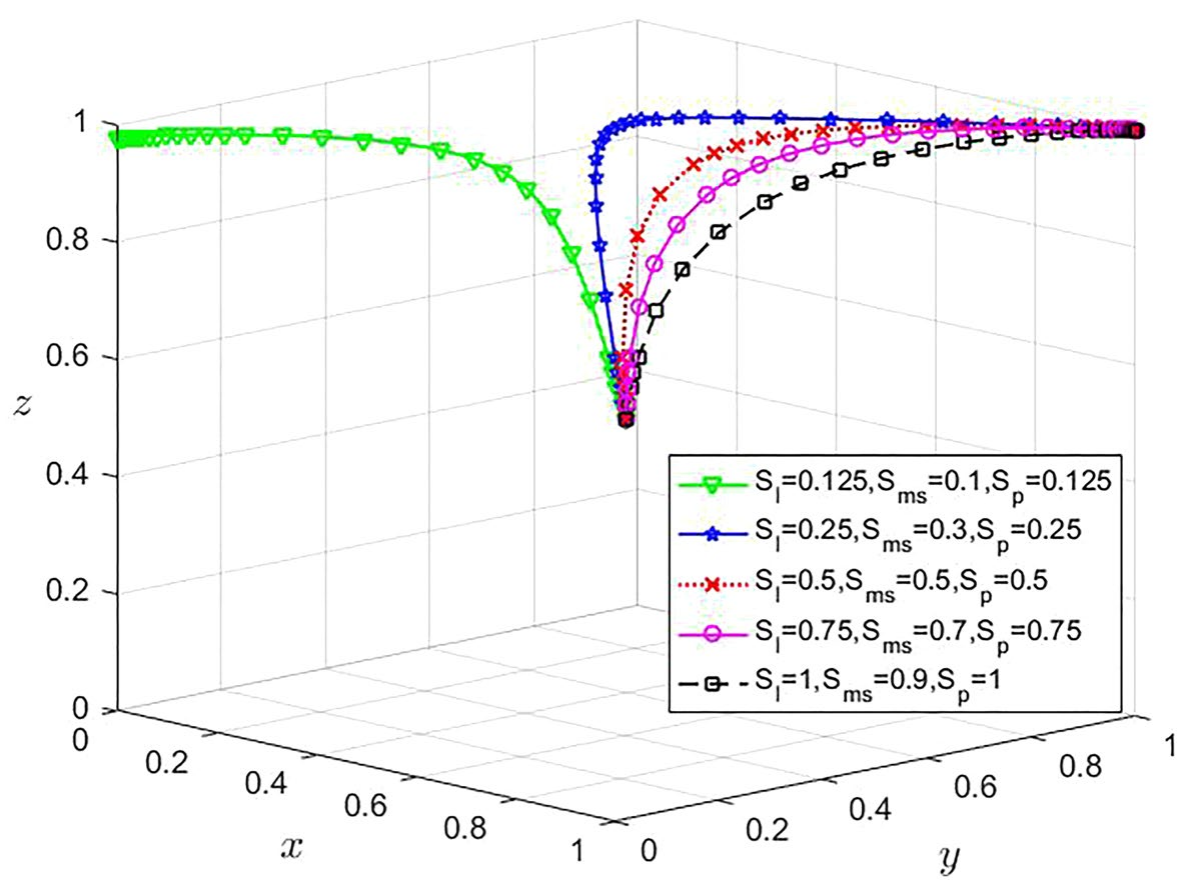
Influence of government subsidies on the strategy selection of the tripartite evolutionary game.

Under the condition that the cost of the enterprise and the platform remain unchanged, in order to analyze the influence of the change of the government subsidy intensity on the strategy choice of the game subject, set ${S}_{l}$ are 0.125, 0.25, 0.5, 0.75 and 1, and the values of ${S}_{ms}$ are 0.1, 0.3, 0.5, 0.7 and 0.9, respectively. ${S}_{p}$ are 0.125, 0.25, 0.5, 0.75 and 1, respectively, and the remaining parameters remain unchanged. The evolution results are shown in Fig. 6.

It can be seen from Fig. 6 that when ${S}_{l}$ ≥ 0.25, ${S}_{ms}$ ≥ 0.3 and ${S}_{p}$ ≥ 0.25, x, y and z all converge to 1, and the ${S}_{l}$, ${S}_{ms}$ and ${S}_{p}$ greater, the faster the convergence speed is; When ${S}_{l}$ ≤ 0.125, ≤ 0.1 and ${S}_{ms}$ ≤ 0.125, x and y all converge to 0, and z still converges to near 1. This may be because the government subsidies not sufficient to cover the risks and costs associated with enterprise data sharing.

Large enterprises and SMEs need to pay a certain cost for data sharing, and there are potential risks of data privacy leakage and data misuse. Consequently, they prefer not to share data when government subsidies are not sufficient to cover these costs and risks. Despite this, cooperation with enterprises is an important way for platforms to gain revenue. Platforms, perhaps with a more strategic foresight, are willing to invest resources to foster enterprise data sharing, as it can augment their user base and data flow, hereby positively impacting their long-term growth. Therefore, even with low government subsidies, platforms are still highly willing to cooperate.

### The impact of digital platform service quality on enterprise strategy choice

**Figure 7:**
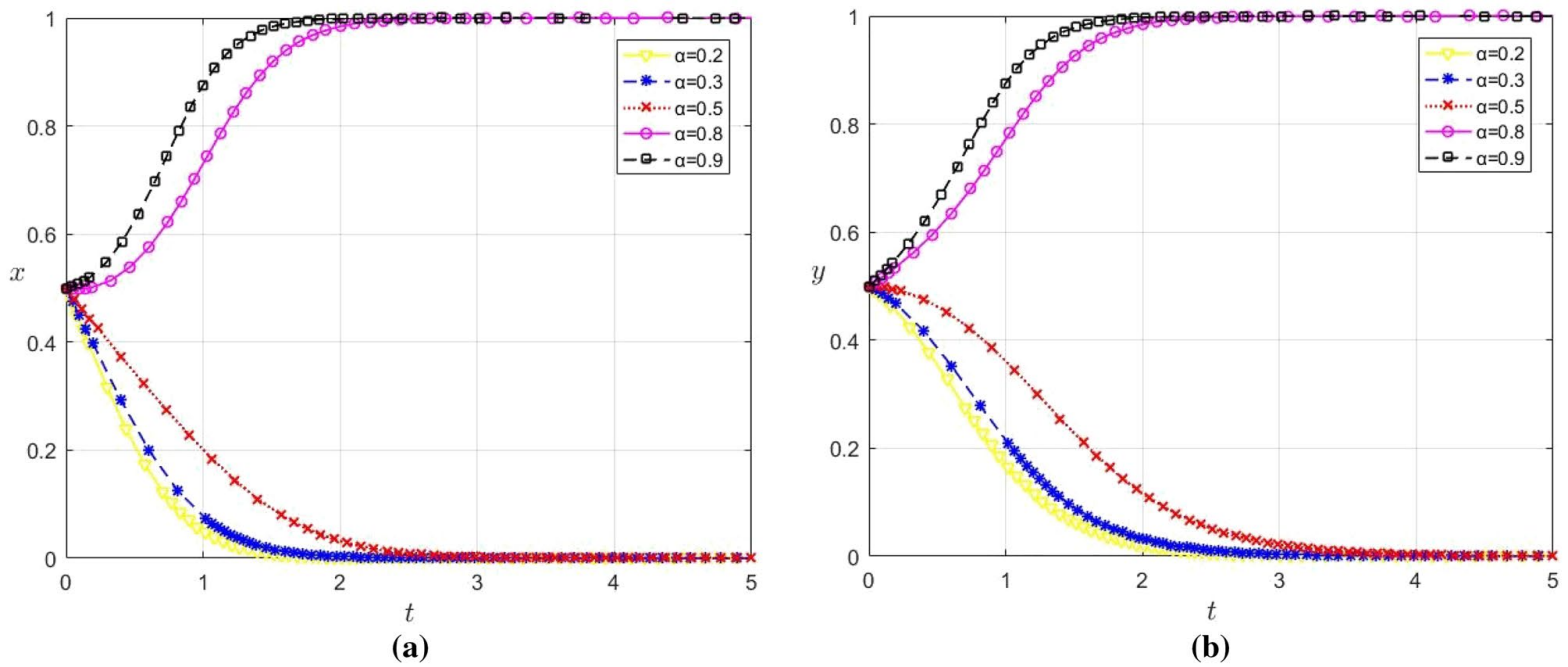
(**a**) Influence of $\alpha$ change on strategy selection of large enterprises (**b**) Influence of $\alpha$ change on strategy selection of SMES.

Since the quality of service provided by the digital platform directly impacts the revenue generated from enterprise data sharing, this paper analyzes the influence of varying service quality on the strategic choices of game players. The values of $\alpha$ are set to 0.2, 0.3, 0.5, 0.8 and 0.9 respectively, and the remaining parameter values remain unchanged. The evolution results are shown in Fig. 7a, b.

According to Figs. 2, 7a and b, when $\alpha$ ≥ 0.7, both x and y converge to 1; When $\alpha$ ≤ 0.5, both x and y converge to 0, with x converging faster than y. Therefore, there exists a certain threshold. When platform’s service quality surpasses this threshold, large enterprises and SMEs will cooperate with platform to share data. This reflects that an increase in the quality of the platform’s services can better meet the needs and expectations of large enterprises and SMEs. If the platform can provide high-quality data processing and analysis services, large enterprises and SMEs are more likely to believe that platform has the ability to utilize the shared data to create greater value for them. Simultaneously, the higher quality of digital platform services, the more sticky benefits platform can gain from cooperation, thereby increasing platform’s incentive to cooperate, as well as the willingness of large enterprises and SMEs to share data.

In addition, it can be found that higher platform service quality has a significant effect on reducing the U-shaped effect in the evolution of large enterprises. This is due to the fact that when platform offers high-quality services, it not only reduces the cost and risk of data sharing for large enterprises, but also facilitates the establishment of long-term cooperative relationships between large enterprises and other SMEs, as well as continuous data sharing. Consequently, it enhances the revenue of large enterprises, thus reducing the U-shaped effect.

### Influence of the improvement degree of data security guarantee framework on the selection of evolutionary game strategies

**Figure 8:**
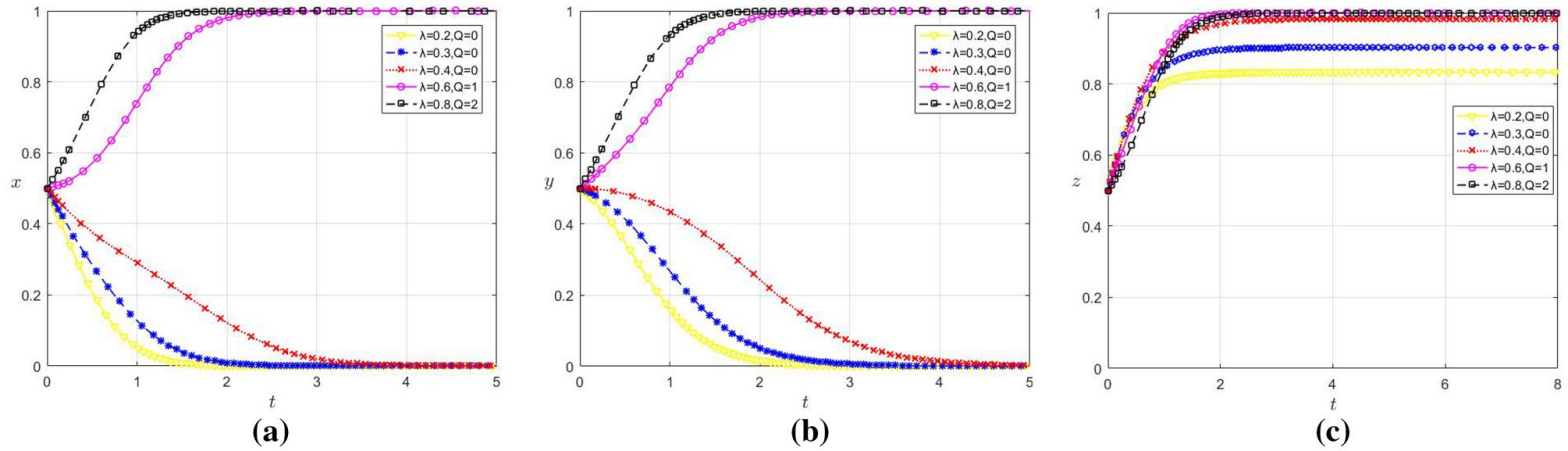
(**a**) Influence of $\lambda$ change on the strategy choice of large enterprises (**b**) Influence of $\lambda$ change on the strategy choice of SMEs c Influence of $\lambda$ and $Q$ change on the strategy choice of platform.

In order to analyze the influence of changes in the improvement degree of data security assurance framework on the strategy selection of each game player, set the values of $\lambda$ are 0.2, 0.3, 0.4, 0.6, 0.8 respectively, and when $\lambda$ < 0.5, $Q$ = 0; When $\lambda$ = 0.6 and $\lambda$ = 0.8, the corresponding values of $Q$ are 1 and 2 respectively, and the remaining parameter values remain unchanged. The evolution results are shown in Fig. 8a–c.

It can be seen from Fig. 2, 8a–c that when $\lambda$ ≥ 0.5, x, y and z all converge to 1, with an increased value of $\lambda$ correlating to a faster convergence rate. Conversely, for $\lambda$ ≤ 0.4, both x and y converge to 0, while z gradually evolves to around 0.8 as $\lambda$ decreases. Therefore, there is a certain threshold, and when the degree of perfection of the data security guarantee framework exceeds this threshold, enterprises will positively engage in data sharing, and digital platforms will choose to cooperate with them.

This phenomenon reflects that when the government can provide reliable data security, it can reduce the risk of data privacy leakage and thus promote data sharing among enterprises. At the same time, the government’s legal policies will strengthen the regulation of digital platforms, prompting them to strengthen their data security capabilities and reduce inherent data risk, so enterprises will trust platforms more and be willing to share data through cooperation with them. Although the technical cost of platforms increased due to the continuous improvement for data security, platforms can still benefit from cooperation with enterprises in the long-term, so them will choose to cooperate with enterprises. Below this threshold, however, the heightened risk of data privacy leakage deters data sharing, especially among large enterprises that, relative to SMEs, hasten to withhold data due to substantial potential losses.

In addition, the refinement of the data security guarantee framework has a positive effect on reducing the U-shaped effect of large enterprises. This is because a comprehensive data security guarantee framework can help large enterprises greatly reduce the security risks associated with data sharing, protect their interests and thus dispelling their apprehensions regarding data risks, effectively alleviating their U-shaped effect in data sharing.

**Figure 9:**
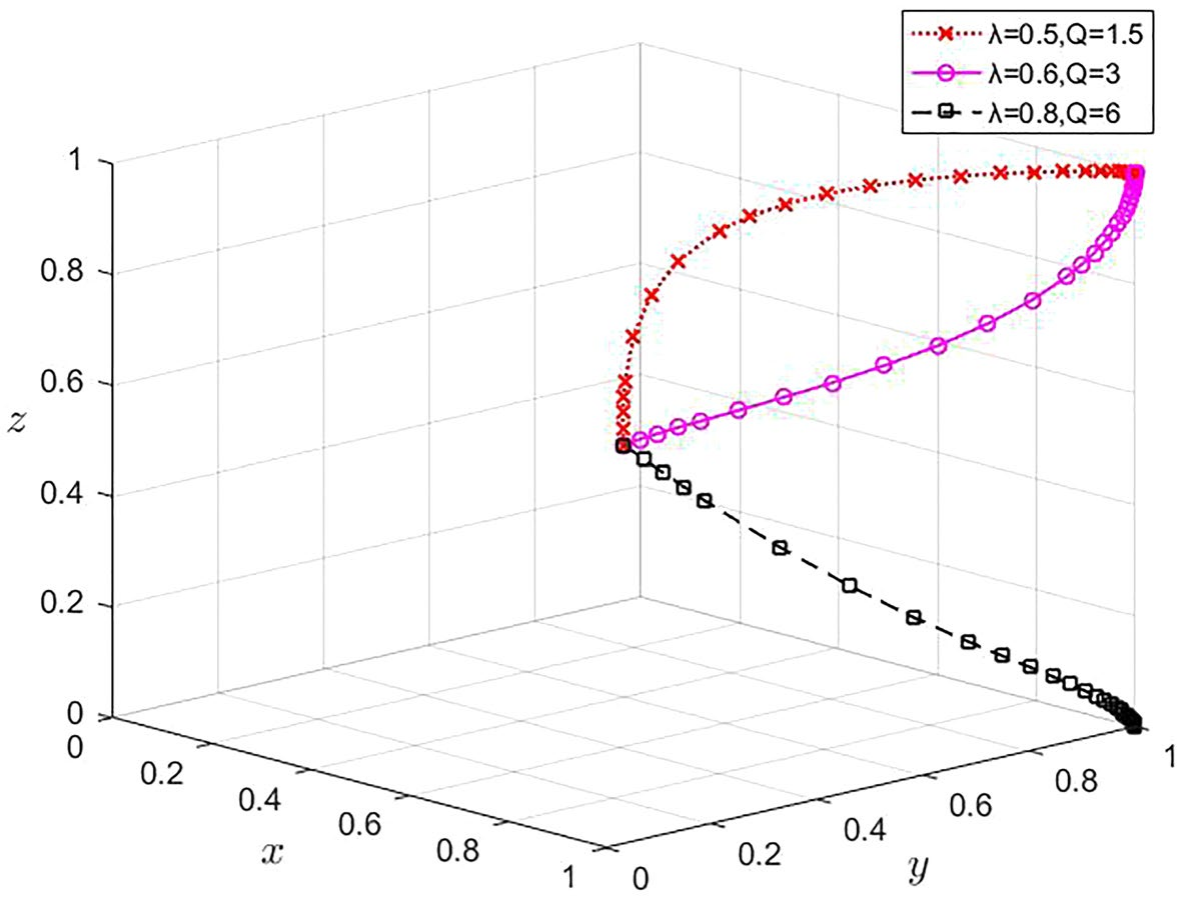
Influence of $\lambda$ and $Q$ changes on the strategy selection of the players in the game.

However, when the values of $\lambda$ are respectively 0.5, 0.6 and 0.8, and the corresponding values of $Q$ are respectively 1.5, 3 and 6, the remaining parameter values remain unchanged, and the evolution results are shown in Fig. 9. When $\lambda$ = 0.8 and $Q$ = 6, although x and y converge to 1, z converges to 0. This is because, as the data security guarantee framework continues to improve, digital platforms will choose not to cooperate when the costs they have to pay exceed what they can afford. However, at this time, as the risk of data leakage is greatly reduced, data sharing through traditional methods can still be beneficial, so large enterprises and SMEs will choose to share data and cooperate.

### Influence of loss caused by information asymmetry on strategy choice of enterprises

**Figure 10:**
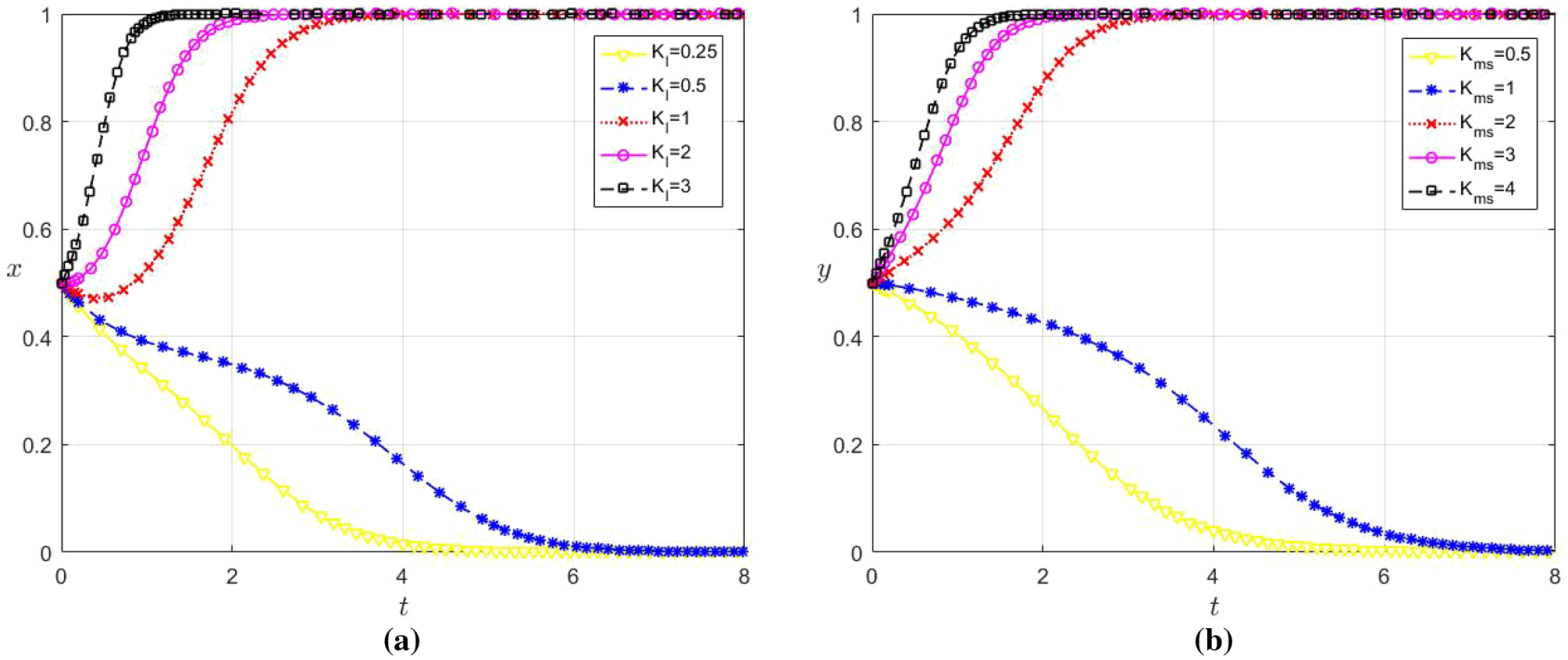
(**a**) Influence of ${K}_{l}$ change on the strategic choice of large enterprises (**b**) Influence of ${K}_{ms}$ change on the strategic choice of SMEs.

In order to analyze the influence of the loss caused by information asymmetry on the strategy choice of the game players, let the values of ${K}_{l}$ are 0.25, 0.5, 1, 2 and 3 respectively; ${K}_{ms}$ are 0.5, 1, 2, 3, 4, and the remaining parameters remain unchanged. The evolution results are shown in Fig. 10a, b.

As observed in Fig. 10a, b, for values of ${K}_{l}$ ≥ 1 and ${K}_{ms}$ ≥ 2, both x and y converge to 1, and the greater, the ${K}_{l}$ and ${K}_{ms}$ faster the convergence speed. Conversely, when ${K}_{l}\hspace{0.17em}$≤ 0.5 and ${K}_{ms}\hspace{0.17em}$≤ 1, both x and y converge to 0. Therefore, for large enterprises and SMEs respectively, there exists a threshold below which large enterprises and SMEs against data sharing when their losses from information asymmetry are less than this threshold. This reflects the fact that large enterprises and SMEs may choose not to share data due to higher cost and data risk considerations when their losses from information asymmetry are relatively low, as such sharing does not mitigate losses but rather augments costs. However, once the loss attributable to information asymmetry exceeds this threshold, there is a heightened incentive for data sharing among enterprises, with SMEs displaying a marginally higher motivation compared to large enterprises.

This phenomenon reflects that when large enterprises and SMEs incur larger losses due to information asymmetry, they may realize that has an increasing impact on their benefits. In this case, in order to minimize losses and increase competitiveness, large enterprises and SMEs may more positively choose data sharing strategies as a way to obtain more information. Additionally, considering the disparities between SMEs and large enterprises in terms of capabilities and resource availability, SMEs may be more reliant on external resources and exhibit a lower tolerance for the risks associated with information asymmetry. Therefore, SMEs are more willing to engage in data sharing than large enterprises.

## Results

This study employs evolutionary game theory to investigate the strategic interactions among large enterprises, SMEs and digital platforms. It analyzes the factors influencing the system’s evolution towards an ideal state under varying parameter conditions, leading to the following conclusions.

1. Due to the large risk loss of data sharing, a U-shaped effect is observed in large enterprises, leading to a temporary decline in their enthusiasm for data sharing. The enhancement of platform service quality and the continuous improvement of data security guarantee framework, significantly mitigates the U-shaped effect.
2. Government subsidies have a positive incentive and moderating effect on data sharing for platform-enabled enterprises. When enterprises or platforms face significant cost pressures, augmenting government subsidies can facilitate tripartite cooperation and realize data sharing. Compared with platforms, enterprises are more sensitive to changes in subsidies, while platform strategy choices are more influenced by costs.
3. Data security guarantee framework is a critical factor affecting the realizing of data sharing. When the framework reaches a certain level, enterprises will still seek opportunities to share data, even in the absence of platforms cooperation. Additionally, the improvement of platforms internal security and service quality, and the reduction of costs of enterprises, can promote enterprises to share data through platforms. Furthermore, the data sharing enthusiasm of SMEs is higher than large enterprises. The loss caused by information asymmetry will also affect the decision of enterprises.

## Discussion

In this study, under the perspective of platform empowerment, by constructing an evolutionary game model between large enterprises, SMEs and digital platforms, analyzing the influence of multiple factors—such as cost and data quality—on the strategic choices of each game subject, and simulating the equilibrium point of the system’s evolutionary stabilization strategy under the simulation of different influencing factors with the use of MATLAB software. This study investigates the evolutionary path of digital platforms empowering large enterprises and SMEs to share data and the realization mechanism.

The findings of this paper confirm some of the previous literature, for example, data risk is a key factor affecting the willingness of enterprises to share data^63,64^, and the application of digital security technology is conducive to reducing the risk of data privacy leakage, which is conducive to the promotion of data sharing^65^. Additionally, the study indicates that digital platforms can positively impact on enterprise data sharing, and this finding is also generally consistent with the viewpoints mentioned in the previous outlook on future enterprise data sharing^66^.

Moreover, this paper presents some original and interesting findings. Firstly, there is a “U-shaped” effect where the willingness to share data initially decreases and then increases among large enterprises, potentially due to the greater data risks associated with data sharing within this enterprise size. Secondly, SMEs are more positive than large enterprises in data sharing, probably because SMEs usually have more limited resources and capabilities; sharing data with large enterprises can gain more business resources and market share, thus enhancing their competitiveness and realizing better development. Finally, large enterprises and SMEs may engage in data sharing even without the empowerment of digital platforms, but only if the government improves the data security framework to create a secure and reliable environment for inter-enterprise data sharing.

Based on the above results and findings, and in conjunction with the current state of global digital economy progress, the following countermeasures are proposed.

1. Strengthen the role of government support and guidance, and enhance the level of regularized supervision. Governments can flexibly formulate subsidy incentive policies based on the different characteristics and roles of enterprises and platforms; strengthen guidance for the cooperative development of large enterprises and SMEs, promote the establishment of a data sharing mechanism, and push forward the in-depth integration of the whole industrial chain. Concurrently, it should accelerate the improvement of the data sharing and protection management frameworks, cultivate and grow the security industry, guarantee data safety and promote the sustainable development of the digital economy.
2. Enterprises should raise awareness of cooperative digital transformation and activate the value of data elements. Large enterprises and SMEs should capitalize on their unique strengths, integrate and innovate development, and jointly improve the modernization level of the industrial chain; heighten their awareness of data as an asset and its security, and refine the data security management hierarchy. Simultaneously, they should strengthen cooperation with digital platforms, enhancing their capacity to integrate and leverage both internal and external data, release the potential value of data, and reduce the asymmetry of information through the complementary sharing of data resources to achieve cost decreasing and benefit increasing.
3. Give full play to the data-driven empowerment function of digital platforms and grow the scale of platform economy. Platforms should augment their technological innovation prowess, tackle core data security technologies, and ensure the safety of data circulation. They also should intensify cooperation and knowledge exchange among platforms and between platforms and enterprises, creating an interconnected platform ecosystem. Furthermore, platforms should develop transformative products and services tailored to enterprise needs, continually refining data analysis and application skills to attract businesses to join and utilize the platform, thus expanding its market scope and value.

## Conclusion

As entering the digital economy, data has become the key link driving the integration between large enterprises and SMEs. It is imperative to study how to promote data sharing for value creation among large enterprises and SMEs. By constructing an evolutionary game model to explore the dynamic evolution process and steady state of platform-enabled data sharing among large enterprises and SMEs under the interaction of different influencing factors, so as to discover the mechanism of data sharing among large enterprises and SMEs. This study finds that cost is the key factor affecting the stability of tripartite cooperation, in addition to improving the service quality of platforms, increasing government subsidies, and strengthening data security protection are all conducive to the promotion of data sharing among large enterprises and SMEs. In the case of data sharing, SMEs and platforms are more motivated than large enterprises, while large enterprises have a U-shaped dynamic change of initial decline followed by an increase motivation in the process.

The paper makes three main theoretical contributions. Firstly, it introduces evolutionary game theory into the research on data sharing, considering the dynamics and decision-making games of digital platforms, large enterprises, and SMEs. This provides a new analytical framework and theoretical perspective for analyzing enterprises data sharing. Secondly, it explores the application of evolutionary game theory in the context of the digital economy, expanding its potential fields of application. Finally, by analyzing the role of digital platforms in promoting data sharing among large enterprises and SMEs, it reveals the internal mechanism of platform empowerment and supports improvements in platform economy and related theories.

The pragmatic significance of this paper is mainly to promote the construction of data sharing and co-development ecosystem between large enterprises and SMEs. On the one hand, it can provide suggestions for the government to formulate data sharing policies and enterprises to choose data sharing strategies. On the other hand, it helps the platform to optimize its service model and fully release the empowerment effect. So as to enhance the attractiveness and user stickiness, and promote the platform’s economic development.

However, this paper also has certain research limitations. Firstly, this paper does not consider the possibility of opportunistic behavior among enterprises, wherein some may exploit data shared by others but not share their own data. If there is no effective punishment mechanism or it is not strong enough, such behavior could proliferate, discouraging data sharing and ultimately undermining the cooperative mechanism. Secondly, although this paper sets numerical simulation parameters by referring to real cases to improve the results reliability, the referenced cases may not represent all situations in reality. Since there may be significant differences in data sharing among enterprises in different industries, the research results may still have some deviations. In addition, the study has not fully considered the long-term impact of changes in the external environment on data sharing, which may change over time, thus affecting the strategic choices and evolutionary paths of data sharing between large enterprises and SMEs.

Against the above research limitations, future research can delve deeper and expand from multiple perspectives. Firstly, future research can consider the impact of opportunistic behavior on data sharing between large enterprises and SMEs. It is important to study how opportunistic behavior affects the willingness of enterprises to share data, and design reasonable reward and punishment mechanisms to circumvent it as much as possible, so as to promote data sharing among enterprises. Secondly, future research can collect a wider range of data and construct a more comprehensive model by analyzing multiple typical cases to improve the authenticity and generalizability of the research results. And we can construct econometric models for empirical analysis by collecting data from different industries to explore the differences in data sharing among different industries, then further refine the mechanisms of data sharing among large enterprises and SMEs empowered by digital platforms. Moreover, future research can focus on how the external environment affects the dynamic evolution of data sharing, such as the changes in policies, so as to propose more targeted and adaptive strategies. Through these efforts, future research will be able to further enrich and develop the research’s content, offering deeper and comprehensive theoretical support and practical suggestions for data sharing among large enterprises and SMEs.

## Data Availability

All data generated or analysed during this study are included in this published article [and all data are available from the corresponding author upon reasonable request].
